# Intralesional deoxycholic acid: A potential therapeutic alternative for the treatment of lipomas arising in the face

**DOI:** 10.1016/j.jdcr.2021.04.037

**Published:** 2021-05-26

**Authors:** Marely Santiago-Vázquez, Eduardo A. Michelen-Gómez, Dianne Carrasquillo-Bonilla, Osward Y. Carrasquillo, Alma Cruz

**Affiliations:** a Department of Dermatology, University of Puerto Rico–School of Medicine, San Juan, Puerto Rico; b University of Puerto Rico–School of Medicine, San Juan, Puerto Rico

**Keywords:** clinical case, cosmetic dermatology, general dermatology, intralesional deoxycholic acid injection, lipoma treatment, lipomas, surgery

## Introduction

Lipomas are common benign tumors of mature fat cells. Although most lipomas are asymptomatic, patients often seek their removal for cosmetic reasons. The current treatment modalities, including surgical excision and liposuction, are highly effective but have a high risk of scarring. When occurring in cosmetically sensitive areas such as the face, noninvasive treatment modalities should be considered to minimize the risk of scarring associated with traditional treatment options. Intralesional injections of deoxycholic acid are currently approved by the US Food and Drug Administration for the removal of excessive submental fat, and the use of this compound for the treatment of lipomas is mainly off-label, with only a handful of cases documenting such use having been reported in the literature.^1^ We report the successful use of intralesional deoxycholic acid in the treatment of a facial lipoma, in which the clinical resolution was achieved with no need for surgery.

## Case report

**Fig 1:**
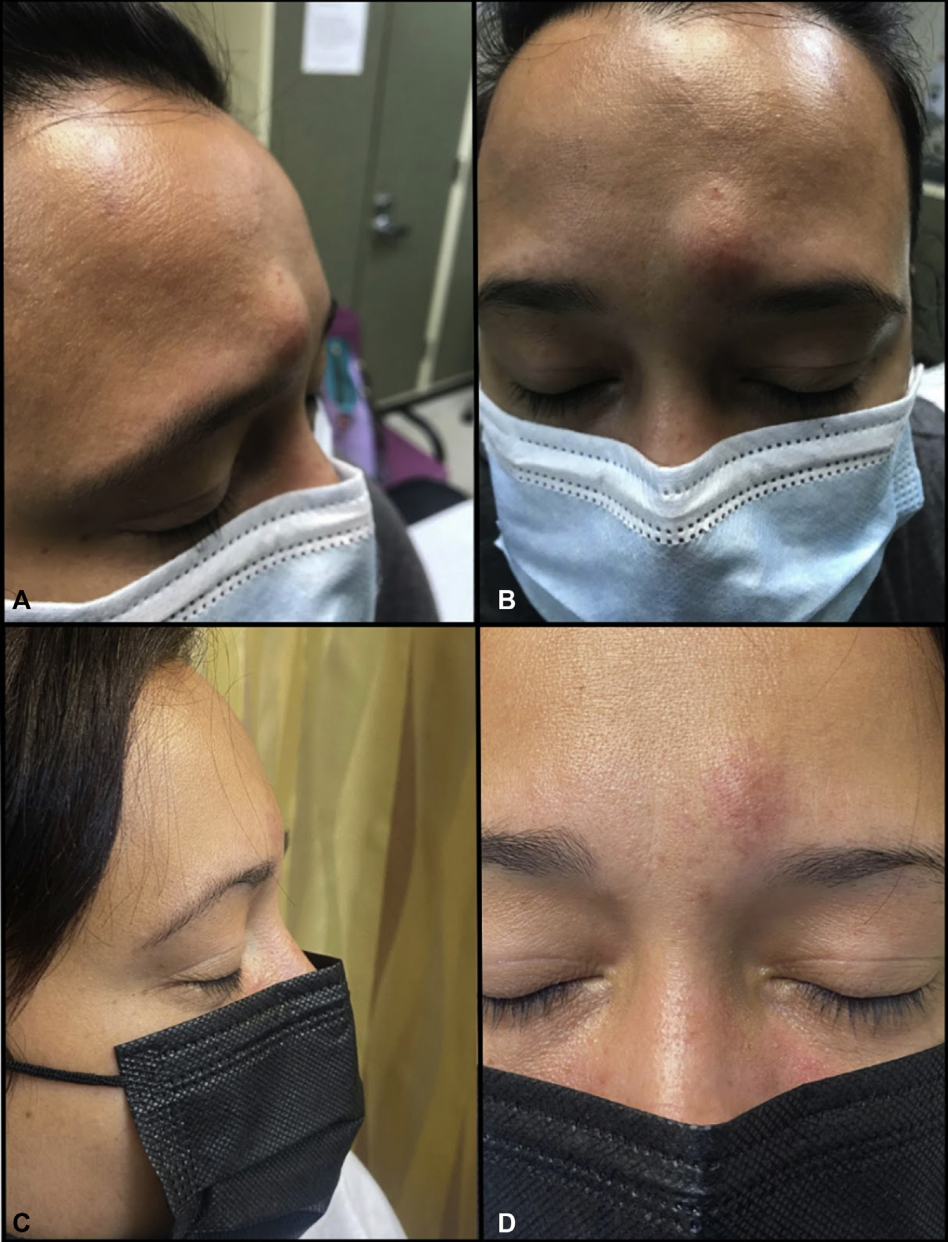
Baseline pretreatment evaluation. **A** and **B**, A 2.0 × 1.8 × 0.7-cm soft, nontender, mobile subcutaneous mass with overlying erythema and telangiectasia located on the right, mid lower portion of the forehead. **C** and **D**, Follow-up evaluation at week 32 after the treatment showed significant clinical improvement with the lesion measuring 0.6 × 0.8 × 0.1 cm.

A 33-year-old Hispanic woman presented with a slow-growing, painless facial lump that was causing cosmetic distress. Initial evaluation revealed a 2.0 × 1.8 × 0.7-cm soft, nontender, mobile subcutaneous mass with overlying erythema and telangiectasia (Fig 1, *A* and *B*) in the right, mid lower portion of the forehead. Ultrasound imaging was remarkable for hypoechoic subcutaneous mass with a few weak internal echoes. Physical and sonographic evaluation of the lesion was consistent with subcutaneous lipoma. Given its appearance, our patient requested that the lesion be excised. However, because of concerns of potential scarring associated with traditional treatment modalities, the patient opted for the off-label use of intralesional injections of deoxycholic acid. Our patient received—at intervals of 3 to 8 weeks—a total of 6 injections, reaching a total cumulative dose of 1 mL. During each treatment, 0.1 mL or 0.2 mL of a 10 mg/mL solution was evenly injected into the middle of the tumor using a 30-gauge needle. Our patient tolerated the treatment well without severe complications, experiencing only a mild burning sensation after the treatment, which resolved within hours. After the second treatment, a reduction in the size of the lesion was noted, and within 14 weeks, the lesion's size was reduced by 50%. The reduction in tumor size continued to be observed during the follow-up injections. On average, there was a 12.5% reduction in the mass size between treatments. At week 32 after the initiation of the treatment, the lesion measured 0.6 × 0.8 × 0.1 cm with only mild residual erythema, telangiectasia, and fibrofatty consistency (Fig 1, *C* and *D*). Given that the patient was satisfied with the results, further treatment with surgical excision was deferred.

## Discussion

Deoxycholic acid is a bile acid with detergent properties; its application results in the emulsification of fat.^1^^,^^2^ Although most commonly used for nonsurgical body contouring, its use for the treatment of lipomas has been gaining attention, with only a few cases having been reported.^3^, ^4^, ^5^, ^6^ However, most of those cases involved lipomas on the trunk. To our knowledge, this is the first case in the literature to describe the successful treatment (ie, the achievement of a cosmetically acceptable result) of a facial lipoma with intralesional deoxycholic acid. Several injection site reactions have been reported in the literature, including swelling, bruising, pain, numbness, redness, skin tightness, and nerve injury. However, our patient only experienced a postinjection mild burning sensation with a prompt resolution. Our case highlights the potential of intralesional lipolysis with deoxycholic acid as a safe and effective therapeutic option for lipomas, especially those occurring on cosmetically sensitive anatomic regions such as the face. However, the need for further studies assessing the role of deoxycholic acid in lipoma management remains.

## Conflicts of interest

None disclosed.
